# Gene-metabolite annotation with shortest reactional distance enhances metabolite genome-wide association studies results

**DOI:** 10.1016/j.isci.2023.108473

**Published:** 2023-11-14

**Authors:** Cantin Baron, Sarah Cherkaoui, Sandra Therrien-Laperriere, Yann Ilboudo, Raphaël Poujol, Pamela Mehanna, Melanie E. Garrett, Marilyn J. Telen, Allison E. Ashley-Koch, Pablo Bartolucci, John D. Rioux, Guillaume Lettre, Christine Des Rosiers, Matthieu Ruiz, Julie G. Hussin

**Affiliations:** 1 Département de Biochimie et de Médecine Moléculaire, Université de Montréal, Montréal, QC, Canada; 2 Montreal Heart Institute, Montréal, QC, Canada; 3 Division of Oncology and Children’s Research Center, University Children’s Hospital Zurich, University of Zurich, Zurich, Switzerland; 4 Department of Pediatric and Adolescent Oncology, Gustave Roussy Cancer Center, Université Paris-Saclay, Villejuif, France; 5 Duke Molecular Physiology Institute, Duke University Medical Center, Durham, NC, USA; 6 Division of Hematology, Department of Medicine, Duke University Medical Center, Durham, NC, USA; 7 Université Paris Est Créteil, Hôpitaux Universitaires Henri Mondor, APHP, Sickle cell referral center – UMGGR, Créteil, France; 8 Université Paris Est Créteil, IMRB, Laboratory of excellence LABEX, Créteil, France; 9 Département de Médecine, Université de Montréal, Montréal, QC, Canada; 10 Département de Nutrition, Université de Montréal, Montréal, QC, Canada

**Keywords:** Association analysis, Computational bioinformatics, Quantitative genetics

## Abstract

Metabolite genome-wide association studies (mGWAS) have advanced our understanding of the genetic control of metabolite levels. However, interpreting these associations remains challenging due to a lack of tools to annotate gene-metabolite pairs beyond the use of conservative statistical significance threshold. Here, we introduce the shortest reactional distance (SRD) metric, drawing from the comprehensive KEGG database, to enhance the biological interpretation of mGWAS results. We applied this approach to three independent mGWAS, including a case study on sickle cell disease patients. Our analysis reveals an enrichment of small SRD values in reported mGWAS pairs, with SRD values significantly correlating with mGWAS p values, even beyond the standard conservative thresholds. We demonstrate the utility of SRD annotation in identifying potential false negatives and inaccuracies within current metabolic pathway databases. Our findings highlight the SRD metric as an objective, quantitative and easy-to-compute annotation for gene-metabolite pairs, suitable to integrate statistical evidence to biological networks.

## Highlights

• The shortest reactional distance (SRD) metric is a useful tool
• Annotating gene-metabolite pairs at various p -values thresholds from mGWAS
• Identifying false negative associations based on small SRD
• Identifying gaps of knowledge in current metabolic pathways

## Graphical abstract

**Figure:**
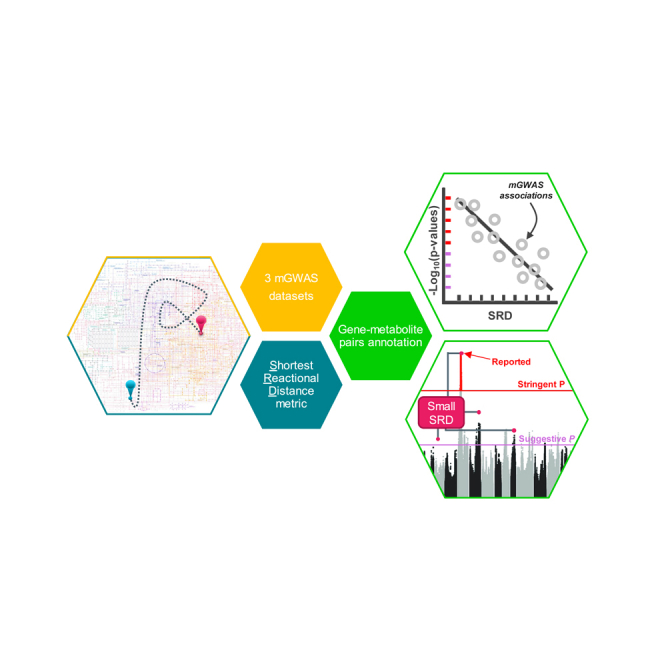

## Introduction

High throughput biotechnologies and analytic approaches applied to large human cohorts have recently revolutionized biomedical research, allowing the quantification and characterization of biological molecules to generate “omics” datasets. Genomics, the characterization of an individual’s DNA molecules, led to the identification of thousands of genetic variants associated with a trait, disease, or response to treatments, through large-scale genome-wide association studies (GWAS).^1^ Although GWAS have resulted in a better understanding of disease mechanisms, these approaches only consider genetic variation established at birth, and ignore the environment of an individual, influencing its biological state. An alternative strategy to complement traditional GWAS and better understand human biology is to comprehensively interrogate disease states at the molecular level using metabolomics, which offers a robust way to systematically measure thousands of low-molecular-weight compounds, called metabolites. After tissue extraction or collection of biological samples (usually blood and urine), metabolites can be detected, identified, and quantified using either mass spectrometry (MS) or nuclear magnetic resonance (NMR).^2^ As biomarkers of the underlying molecular dysfunctions, metabolite levels correspond to intermediate phenotypes (or endophenotypes) representing natural or clinical heterogeneity. Storing and sharing metabolomics knowledge is the focus of the Human Metabolome Database (HMDB), which is one of the largest and comprehensive curated collection of human metabolite and human metabolism data in the world.^3^

Metabolomics can be seen as the study of the ultimate molecular response of an organism to genetic, environmental, and pathological modifications, but elucidating specific molecular mechanisms from metabolomics alone is difficult since many metabolites are involved in multiple biological processes. Linking metabolomics signals with genetics provides a promising approach to identify the implicated pathways and, as such, metabolite genome-wide association studies (mGWAS) are key approaches to integrate metabolomics with genomics. mGWAS report associations between metabolites and genetic loci, referred to as metabolite quantitative trait loci (mQTL), making it possible to study associations between millions of genetic variants and thousands of metabolites and to generate insightful hypotheses about uncharacterized regulatory mechanisms.^2^ Although very powerful, interpretation of mGWAS results remains challenging. Indeed, millions of statistical tests are generally performed between single nucleotide polymorphisms (SNPs) and metabolites, leading to a huge multiple testing burden.^4^ Furthermore, interpreting the biological meaning of an association between a given SNP and a metabolite requires interdisciplinary knowledge. In this context, the simplest way of prioritizing hypotheses remains to apply a conservative correction for multiple testing such as Bonferroni or Benjamini-Hochberg procedures.^4^ The consequence of this correction is that only associations with the most significant p values are reported whereas biologically relevant associations with a suggestive p value may be missed.

With the increasing number of published mGWAS and their current limits, it is necessary to develop systematic methodologies to gain better insight into the mGWAS results by exploiting the most up-to-date biological knowledge. There are multiple resources available that aim at storing and describing known relationships between genes and phenotypes or endophenotypes (GWAS Catalog,^5^ PhenoScanner,^6^ OpenGWAS,^7^ Open Targets Genetics,^8^ PheLiGe,^9^ and DisGeNET^10^) as well as tools to annotate gene-metabolites pairs based on the available resources but none of them have been specifically designed to address current mGWAS limitations.^11^^,^^12^ Only few methods have been developed to gain insight into the biological interpretation of mGWAS data specifically,^13^^,^^14^ and none have been explicitly used to annotate mGWAS results based on well-curated biochemical knowledge, such as the Kyoto Encyclopedia of Genes and Genomes (KEGG) database.^15^ KEGG is recognized for its high curation level of metabolic pathways for many model organisms, including humans, and is a reference for the reporting of enzymatic reactions, which is needed to understand the functional importance of gene-metabolite pairs. KEGG is a reference for the biochemical functions of genes with the descriptions of enzyme reactions, but a systematic and quantitative annotation procedure using this database in the context of mGWAS results remains to be explored. In this regard, recent studies have highlighted the applicability and utility of topological data analysis based on graph theory approaches,^16^ including the shortest path,^17^^,^^18^ in revealing biological knowledge in the context of complex metabolic networks.^13^^,^^17^^,^^18^ For example, the shortest path was used to characterize the impact of gene deletion on nearby metabolites within the metabolic network of *E. coli*.^18^ It was also used to analyze the relationship between expression quantitative trait loci (eQTL) and metabolomic data, for example within the metabolic network of rat adipose tissues.^17^ Still, this type of metric has not been applied to systematically annotate mGWAS results.

In this study, we assessed the utility of the shortest reactional distance (SRD) metric computed from KEGG database’s pathways to annotate mGWAS results and to help extract biological insights from them. We developed PathQuant, an R package, to enable a robust and systematic computation of SRD values between any lists of gene-metabolite pairs mapped onto KEGG graphs, which represent metabolic pathways, while keeping the original, well-curated, topology of each queried pathway. Focusing on genes encoding for enzymes and their associated metabolites, we applied the SRD annotation to two previously published mGWAS datasets in individuals from different ethnicities: an mGWAS^11^ performed on 7,824 participants from the TwinsUK cohort^19^ and the Kooperative Gesundheitsforschung in der Region Augsburg (KORA) study,^20^ referred herein as the TK study, and an mGWAS^21^ performed on 614 Qatari participants from the Hamad Medical Corporation (HMC), referred herein as the HMC study. We explored results at varying levels of statistical significance. We found that the SRD metric enables identification of associations that do not meet currently accepted cut-off of statistical significance (suggestive associations) but which have high biological relevance. Finally, we performed an mGWAS on previously reported genetic and metabolomic data from a sickle cell disease (SCD) cohort,^22^^,^^23^ referred herein as the SCD study, and show how the SRD metric can be used to prioritize novel hits, including hits with a suggestive p value.

## Results

### Overview of study pipeline

**Figure 1:**
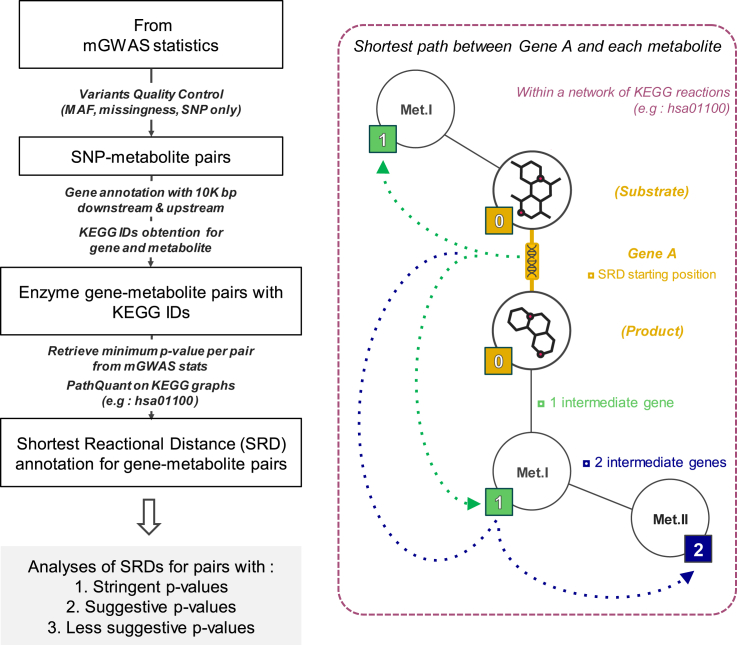
Overview of the Shortest Reactional Distance (SRD) annotation process On the left panel, we show the main steps of the pipeline used to annotate mGWAS datasets. Boxes indicate input and outputs of processing steps described on arrows. The right panel shows the meaning of an SRD annotation of Enzyme A’s pairs within a KEGG pathway, where genes are represented as edges and metabolites as nodes. The SRD between Enzyme A and its main reactants, the substrate and the product, are of 0, everything running deeper adds 1 at each step (dashed arrows): green metabolites are at SRD = 1 and the blue metabolite is at SRD = 2 from Enzyme A.

PathQuant is a tool that converts a metabolic pathway map into a graph of biochemical reactions with metabolites as nodes and genes as edges, to compute the SRD path between a given gene-metabolite pair (STAR Methods, supplemental information, Figure S8). To explore the potential of SRD values as an annotation metric to inform on the biological relevance of gene-metabolite pairs obtained from mGWAS, we developed a pipeline, presented in Figure 1. First, we gather mGWAS summary statistics and perform standard quality-control filters on genetic variants based on minor allele frequencies (MAF) and completeness. The second step of our pipeline only keeps biallelic SNPs at MAF >0.01 (to exclude rare variants) and tested across all measured metabolites, but indels, tri-allelic SNPs and rare variants can be easily integrated, if present in mGWAS summary statistics. Third, SNPs are mapped to their closest genes coding for an enzyme (within 10 kb upstream and downstream) to obtain gene-metabolite pairs. As multiple SNPs will generally be linked with the same gene, only the minimum p value of all SNPs is kept for a gene-metabolite pair, representing the strongest statistical signal for each pair within a study. These p values can be categorized as significant, suggestive, or non-significant according to appropriate thresholds that depend on the dataset (STAR Methods). We then retrieve KEGG IDs from all pairs (STAR Methods), and only retain pairs for which both IDs could be found. Third, we run PathQuant R package to compute the SRD value on all remaining pairs based on the KEGG overview graph (hsa01100). Note that other KEGG graph, or a combination of KEGG graphs, can be easily added at this step. SRD values obtained can be numerical when a path is found, can be given an infinite value (noted as “Inf”) when there is no path between the gene and metabolite in the queried graph, or an “NA” value when the gene or the metabolite (or both) are not found in the queried graph, despite having a valid KEGG ID. We can then perform several analyses of SRD values, as detailed in the following.

### Stringent and suggestive associated pairs have shorter reactional distances

**Figure 2:**
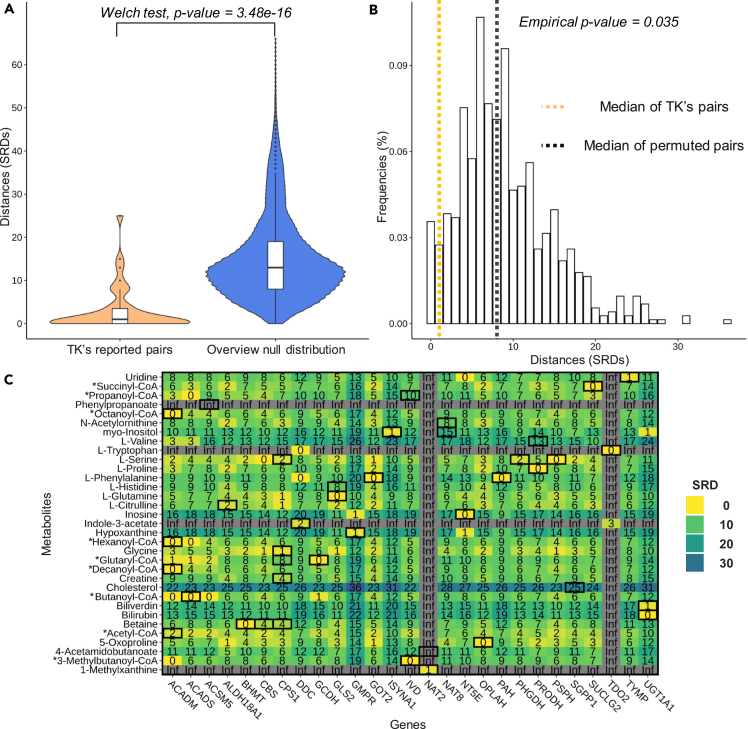
SRD of stringently associated gene-metabolite pairs in the TK study (A) Comparison of SRD annotations for reported genome-wide significant associations from the TK study (orange) and the distribution of all SRD values within KEGG overview graph (hsa01100). (B) Distribution of SRD values computed from permuted gene-metabolite pairs from TK study, with a median SRD of 8 (black dotted line). The median SRD of 1 (orange dotted line) represents an empirical p value of p = 0.035. (C) Heatmap representing all genes and metabolites included in reported genome-wide significant associations from the TK study. The 40 mapped gene-metabolite associations are enclosed in black boxes on the heatmap (n = 40). Abbreviations: CoA = Coenzyme A. ∗ CoA metabolites are proxies for measured carnitines.

To test whether the SRD is a metric capable of capturing the biological relevance of a gene-metabolite pairs, we first explored the results of the TK study, as it represents one of the largest mGWAS studies conducted to date. This mGWAS was done on a group of participants from European descent, and many findings were replicated in follow-up studies, making it a well-validated dataset. Furthermore, it is considered a reference study by the community, resulting in an ideal dataset to test the hypothesis that stringently associated pairs will have lower SRD values. We started to explore this dataset by using the 74 gene-metabolite pairs with KEGG IDs passing the genome-wide significance cut-off set by the original authors^11^ (Table 1). Among these, 40 gene-metabolite pairs were mapped onto KEGG overview graph. After excluding the two pairs with infinite SRD values, the median SRD value for the remaining 38 pairs is 1, which indicates a close biological relationship between the genes and their associated metabolites (Figure 2). To assess how significant this result is, we used two strategies: we compared the SRD values to (1) a null distribution (STAR Methods), built from all possible pairs of gene-metabolites that are present on the KEGG overview graph (Figure 2A); (2) to a permuted set of gene-metabolite pairs (Figure 2B), built with the 33 metabolites and 27 genes involved in the 40 significant gene-metabolite pairs (Figure 2C). There is a statistically significant difference between the TK pairs’ SRD values and the null distribution (Welch test, p value < 3.48 × 10^−16^), confirming the close biological relationship between mGWAS gene-metabolite pairs in the TK study. Similarly, the median SRD value for the permuted pairs is 8, which is significantly higher than the median SRD of 1 (empirical p value = 0.035). These results confirm that closely connected genes and metabolites are enriched in gene-metabolite pairs discovered in mGWAS.

**Table 1:** Dataset description of used dataset for the TK, HMC, and SCD studies

Study name	TK	HMC	SCD	TK-None
Summary stats cut-off	1.03 × 10−10	1.79 × 10-7	None	None
Sample size	7,824	614	651	7,824
SNP-Metabolite name pairs (unique SNP - unique metabolites)	336 (218-186)	6 465 (3 192-100)	3 139 070 208 (24 523 986-128)	1 236 909 025 (2 617 408–486)
Enzyme Gene-Metabolite pairs with KEGG IDs: PathQuant input (unique genes - unique metabolites)[gene - metabolite proportion mapped∗]	74 (43–56)[20%–30%]	68 (32-20)[1–20%]	209 645 290 (3875-118)[16%–92%]	46 779 684 (3815-177)[0.14%–36%]
Pairs’ SRD annotations within KEGG Overview Graph (unique genes - unique metabolites∗∗)[gene - metabolite proportion mapped∗]	SRD: 38 Inf: 2 NA: 34 (27–33)[63%–59%]	SRD: 38 Inf: 4 NA: 26 (13 - 8)[40%–40%]	SRD: 82 145 Inf: 38 980 NA: 336 125 (1275 - 95)[33%–81%]	SRD: 86 219 Inf: 55 822 NA: 533 214 (1257 - 113)[33%–64%]

Line 1: Dataset label for TK, HMC, and SCD studies and the TK dataset without p values cut-off (TK-None). Line 2: Maximum p value cut-off. Line 3: Maximum number of samples available for the mGWAS study. Line 4: Number of SNP-metabolites pairs. Line 5: Number of gene coding for enzyme-metabolite pairs for which KEGG IDs have been obtained. Line 6: Number of SRD annotation within map hsa01100 (KEGG overview graph) categorized in three categories. Lines 4, 5, and 6 have the count of unique genes and metabolites involved in pairs between parenthesis. ∗: the proportion of genes and metabolites mapped from the previous line is indicated between brackets. **∗∗**: the number of unique genes and metabolites are ignoring NA values. Abbreviations: ID = Identifier; SNP = Single-nucleotide polymorphism; SRD = Shortest Reactional Distance; Inf = Infinite value; NA = Not available, a missing annotation.

To visualize the relationship between all genes and metabolites involved in the significant pairs reported in the TK study within KEGG overview graph, we used a heatmap representation of SRD values (Figure 2C), highlighting the reported significant pairs using thick black boxes. Seventeen of these associations have an SRD of 0, implying that these gene-metabolite pairs are from a single enzymatic reaction. For example, PSPH (phosphoserine phosphatase) is catalyzing the formation of L-serine and results in an SRD of 0. Interestingly, within the 40 associations with an SRD annotation, some genes are closely connected to multiple metabolites, such as *ACADM* and *CPS1* that have multiple small SRD values. Moreover, some genes are isolated and appear to be part of disconnected subgraphs of the KEGG overview graph, such as *GMPR* (guanosine monophosphate reductase), *NAT2* (N-acetyltransferase 2) and *TDO2* (tryptophan 2,3-dioxygenase), which obtained infinite SRD values with most metabolites.

The most widely used approach to report mGWAS gene-metabolite pairs is based on genomic distances, but identifying the potential causal gene behind an SNP association is highly challenging. We evaluated the validity of the distance criteria based on SRD. Using the TK dataset, we evaluated the number of additional gene-metabolite pairs and the SRD distribution of pairs when increasing the interval size (10 kb as baseline, 50 kb, 500 kb, 1 Mb) to map the gene (STAR Methods, Figure S3). At an mGWAS p value below 1 × 10^−7^, the 10 kb baseline yielded a total of 76 pairs, and extended intervals incrementally introduced 8, 105, and 70 new pairs for 50 kb, 500 kb, and 1 Mb, respectively. The mean SRD for the 10 kb baseline pairs is 2.6, and 3.2, 7.7, and 9.5, respectively, for the extended intervals (Figure S3A). This suggests that the relevance of inclusion of genes decreases as distance from the mQTL increases. Furthermore, considering the complexity in the regulation of gene expression, protein levels and metabolite levels, other studies have used eQTL annotations in mGWAS studies to define gene-metabolite pairs.^24^ We thus further compared the 10 kb baseline approach to eQTL annotations taken from the eQTL Gen consortium data.^25^ The eQTL annotations alone led to 48 pairs at a mGWAS p value below 1 × 10^−7^, with 28 pairs (58%) already captured in the 10 kb intervals, and 20 new gene-metabolite pairs, involving 14 additional enzymes and 5 additional metabolites. Interestingly, the mean SRD for all eQTL pairs is 7.4, which is comparable to the mean SRD obtained for pairs in the 500 kb interval. Additionally, for the eQTL pairs overlapping the 10 kb and 50 kb intervals, the mean SRD is 4.3 and 5.2, respectively (Figure S3B), suggesting that the eQTL annotation does not necessarily captures relationships in close proximity within metabolic pathways, and supporting the use of both eQTL and SRD annotations for mGWAS.

The design of mGWAS can be heterogeneous, with distinct genomic and metabolomic technologies and pre-processing steps used across studies. To demonstrate the applicability of our annotation more broadly, it is important to use an independent study to replicate the aforementioned observations. Furthermore, there is a widely recognized bias toward white Europeans in genetics studies, which often makes results less generalizable in other ethnicities,^26^^,^^27^ highlighting a need for new bioinformatics solutions to be tested in underrepresented populations. In line with these criteria, we further tested our approach in the HMC study, which has been performed in a different ethnic group, on Qatari individuals. In this mGWAS, the genomic data comes from the exome sequencing technology compared to genotyping data in the TK study, and the metabolomics data are generated using differing pre-processing steps (see STAR Methods). Furthermore, the authors of this study identified SNP-metabolite pairs with suggestive p values, which were made available (Supplementary File number 7 of a study by Yousri et al^21^), allowing us to explore whether including the suggestive mGWAS association results replicated the observation from the TK study. Of 68 gene-metabolite associations (Table 1) at a cut-off of p ≤ 1.4 × 10^−7^, 42 were mapped to the KEGG overview graph for which SRD values were computed. Four pairs had infinite SRD values (see Figure S1C), while the 38 gene-metabolite remaining pairs (Table 1) had SRDs of 0 or 1 (Figure S1), meaning that we have associations that are either substrate-product associations or with one intermediate step. Thus, we replicated the observation seen in the TK study of a significant enrichment of low SRD values compared to the null distribution in this second mGWAS (Welch test, p value < 7.91 × 10^−60^, Figure S1), even when considering a lower significant threshold than standard genome-wide cut-offs.

### SRD annotation can identify false negative hits

**Figure 3:**
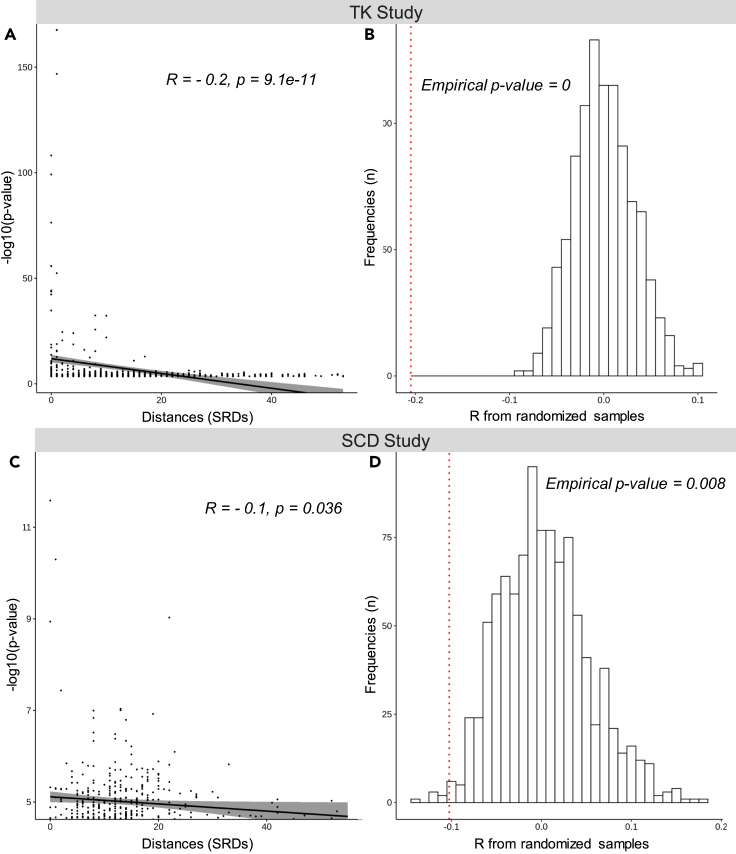
Relationship between p values and SRD values for TK and SCD studies Correlation plot between the –log10(p values) and SRD values for gene-metabolite pairs in (A) the TK study (p value cutoff was 3.16 × 10^−4^, Figure S2B) and (C) the SCD study (p value cutoff was 3.16 × 10^−5^, Figure S2D). Correlation computed on permuted data (N = 1000) to take into account the graph structure allowed computation of empirical p values for TK (B) and SCD (C), with the true correlation coefficient presented (dotted red line).

By using two different studies and different summary statistics cut-offs, we have determined that associations with stringent and suggestive p values are enriched for low SRDs. We next explored more formally the relationship between the SRD values and the p values from the TK study. We graphically defined the gene-metabolite significance cut-off at p < 3.16 × 10^−4^ of the TK study based on a QQplot (Figure S2B, STAR Methods) and used it as a threshold for the minimum value to determine the relationship between SRDs and p values. We observed a significant correlation between the mGWAS p values and SRD values (R = −0.2, p = 9.1 × 10^−11^, Figure 3A), meaning that the higher the significance of an association between a metabolite and a gene is, the closer they are likely to be in the KEGG overview graph. We replicated this correlation using different strategies to map gene-metabolite pairs: based on genomic distances (from 50 kb to 1 Mb, Figure S4), and based on eQTL annotations (Figure S5). This negative correlation confirms the enrichment of biologically relevant pairs in significant associations, and suggests that combining p values and SRD may help prioritizing associations of high biological interest. Furthermore, the SRD value could be used to select for further analysis pairs with small SRD having low p value that nevertheless do not pass stringent significance cut-offs.

As a result of multiple testing burden between each variant and each metabolite level, only top hits are generally reported in mGWAS, but it is well known that there could be false negative hits.^28^ Thereby, we tested the potential for the SRD metric to identify some of these false-negative candidates in these published mGWAS. We define a false negative association, an association that was not reported as a hit in the original mGWAS because the p value was above the significance threshold of single-variant p values but was subsequently reported as a hit in external studies. In the TK study, we focused on the genes and metabolites involved in stringent associations (presented in the heatmap, Figure 2C), which include 27 genes and 33 metabolites. We noticed gene-metabolite pairs annotated with a smaller SRD than the initially reported association, such as the *ALDH18A1* (aldehyde dehydrogenase 18 family member A1) and L-citrulline pair, which has an SRD value of 2, whereas other pairs involving L-citrulline show smaller SRD values. For instance, the *CPS1* (carbamoyl-phosphate synthase 1) and L-citrulline pair, which has an SRD = 1, but was not reported as a significant association in the TK study, despite a p value of 1.749 × 10−8. The association between *CPS1* and L-citrulline has however recently been described twice in the literature with a p value of 1 × 10−25 with SNP rs1509820^29^ and 1 × 10^−14^ with SNP rs975530777,^30^ which exemplifies a situation where an association goes unreported because of the stringent p value cut-offs. The SRD value of 1 reflects a sequence of reactions pertaining to the urea cycle catalyzed by CPS1 for the formation carbamoyl phosphate from ammonia and bicarbonate and by the ornithine transcarbamylase (OTC) for the formation of L-citrulline from carbamoyl phosphate and L-ornithine^31^

Using the same approach, we also identified an example within the HMC study of a gene-metabolite pair at SRD = 0, which was not reported as a significant association because of a suggestive p value (p = 1.17 × 10^−7^). This association is between *UMPS* (uridine monophosphate synthetase) and orotate (Figure S1C, indicated in red). UMPS is a bifunctional enzyme that is part of the *de novo* pyrimidine biosynthetic pathway; its orotate phosphoribosyltransferase subunit catalyses the addition of ribose-5-phosphate to orotate to form orotidine monophosphate (KEGG reaction ID R01870). Given that a significant association has been previously reported between *UMPS* gene and orotate,^32^ we could consider that this finding is replicated in the HMC study but only by adding information about its SRD value.

Taken altogether, the examples highlighted the opportunity to explore associations annotated with low SRD values in mGWAS, beyond those with stringent significance cut-offs. Identifying these potential false negative hits illustrates how useful the SRD annotation can be in discovering biologically relevant results which would be missed when only considering stringent p value cut-offs in mGWAS reporting.

### Case study: mGWAS in sickle cell disease patients

To exemplify how the SRD metric can be used to prioritize mGWAS results, we present a new mGWAS analysis performed in SCD patients of African or African American ancestry. While the cause of SCD has been known for over a century, the molecular determinants of the severity of this blood disease remain unknown and are influenced by genetic variants unlinked to the beta-globin gene.^33^ An mGWAS was performed in 651 SCD patients, quantifying a total of 128 metabolites, to identify metabolites associated with genetic markers. A total of 165 unique SNPs (with MAF >1%) passed a genome-wide significance cut-off of p < 7.8125 × 10^−10^ (Figures S2 and 1 × 10^−7^/128 metabolites) and an additional unique 256 SNPs passed a suggestive cut-off of p < 1 × 10^−7^ (Figure S2C). We identified a total of eight loci associated with metabolite levels (Figure S6), with five of them reported in previous studies (Table S2, containing significant associations from the SCD study). The three additional associations were not previously reported neither in the TK/HMC studies, nor in a large mGWAS of human blood metabolites.^34^ In all three cases, the frequency of the top associated SNP is larger in individuals of African descent than in Europeans according to the gnomAD Genomes database, but these hits were not found in the four largest mGWAS done in individuals of African ancestry to date.^35^^,^^36^^,^^37^^,^^38^ We recognize that while interesting, these novel associations will need replication in an independent cohort.

**Figure 4:**
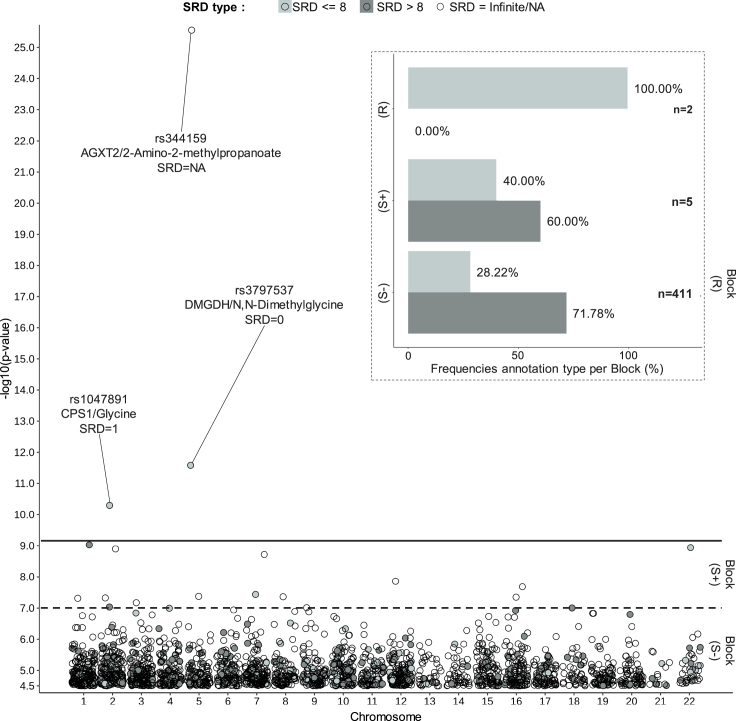
Identification of potential false negative hits for different p values cut-offs in the SCD study The main panel represents -log10(p values) for gene-metabolite pairs for SNPs on each chromosome (x axis). Coloring represents the SRD annotation from 3 categories: SRD = Infinite or NA (white), numerical SRD ≤ 8 (light gray), numerical SRD > 8 (dark gray). On the top right panel, the histogram represents the frequencies of SRD ≤8 and >8 for each block. The frequency percentage is obtained by summing only the pairs with a numerical SRD value (n) within each category according to different p value cut-offs: R: p < 7.8125 × 10^−10^, S+: 7.8125 × 10^−10^ < p < 1 × 10^−7^ and S-: 1 × 10^−7^ < p < 3.16 × 10^−5^.

We generated a QQplot based on minimum p values for the gene-metabolite pairs extracted from the SCD mGWAS (STAR Methods) and defined the gene-metabolite significance cut-off at p < 3.16 × 10^−5^ (Figure S2D). We observed a significant correlation between the mGWAS p values and SRD values (R = −0.1, p = 0.036), replicating the result observed in the TK study, demonstrating that this relationship between significance and SRD is reproducible in a disease cohort, where biological mechanisms can be altered (Figure 3C). Given these observations, we investigated whether associations above the gene-metabolite significance cut-off of p < 3.16 × 10^−5^, but below genome-wide significant cut-offs usually required to report associations, could be prioritized according to SRD values (candidate pairs below the plain line in Figure 4). We split the associations into two suggestively significant categories, one that includes association p values between the genome-wide significance cut-off and p < 1 × 10^−7^ (Figure S2C) and another that includes associations p values between p < 1 × 10^−7^ and the gene-metabolite significance cut-off of p < 3.16 × 10^−5^ (Figure S2D), which we labeled S+ and S-, respectively. To evaluate the potential of association results in each significance categories, we aimed to compute the proportion of small SRD values. From the distribution of all SRD values computed on the KEGG overview graph (STAR Methods), we observed that 25% of SRD values (first quartile) are lower or equal to 8 (Figure S7). This graph-based measure has the advantage of being easily calculated on any KEGG graph available. The first quartile provides an interesting range that captures the cascade of reactions involved in metabolic pathways, enhancing the potential for discovery, and as such provided a valuable range of relatively small distances to detect false negative hits. Furthermore, based on our results, 90% of gene-metabolite significant pairs in the well-powered TK and HMC studies have SRD ≤8 (Figures 2 and S1), and eQTLs supported by mQTL evidence have a mean SRD of 7.4 (Figure S3B). In SCD, out of the genome-wide significant associations with a numerical SRD value in this mGWAS (Block R, Figure 4), two out of three have SRD ≤8. We thus used the SRD value ≤8 as a threshold for considering SRD values as “small”, reflecting the close biological relationships based on the KEGG overview graph topology.

Two (40%) out of five associations from the S+ category have small SRD values, and 116/411 for the S- category (28.22%). Next, we manually investigated the 118 suggestive associations with SRD ≤8 by searching the literature for these gene-metabolite pairs and found six previously described associations (5%): *PRODH* (proline dehydrogenase 1) and L-proline,^11^^,^^39^^,^^40^ *NT5C3A* (5′-nucleotidase, cytosolic IIIA) and orotate,^32^ *GATM* (glycine amidinotransferase) and creatine,^41^ *UPB1* (beta-ureidopropionase 1) and 3-ureidopropionate,^34^ *UGT2B17* (UDP glucuronosyltransferase family 2 member B17) and sn-glycerol 3-phosphate (previously described for total phosphoglycerides),^42^ *DGKH* (diacylglycerol kinase eta) and tetradecanoyl-carnitine,^43^ implying that these are likely to be real associations in SCD patients (Table 2). We thus estimate that at least 5% of associations in this category are false negative and that the SRD metric has added value in identifying them. It also demonstrates the ability of the SRD metric to retain pairs that would not be otherwise reported, thus improving the mGWAS’ potential for discovery.

**Table 2:** Associations with SRD lower or equal to 8 for the SCD study, illustrating potential false negative hits discovered by manual verification

SNP(Chromosome_Position_Allele1_Allele2)	Gene symbol (KEGG ID)	Metabolite common name (KEGG ID)	p value	SRD	Comments – Publication ID reporting the described association
22_18905964_C_T	PRODH(hsa:5625)	L-Proline (C00148)	4.774e-06	0	rs2904552, PMID:24816252, 26068415, 25569235http://www.phenoscanner.medschl.cam.ac.uk/?query=PRODH&catalogue=mQTL&p=1e-5&proxies=None&r2=0.8&build=37
7_33081514_A_G	NT5C3A (hsa:51251)	Orotate (C00295)	3.659e-08	2	rs4316067, PMID: 23823483http://www.phenoscanner.medschl.cam.ac.uk/?query=NT5C3A&catalogue=mQTL&p=1e-5&proxies=None&r2=0.8&build=37
15_45682944_T_C	GATM (hsa:2628)	Creatine (C00300)	5.160e-06	1	rs536148271, PMID: 34226706, http://www.phenoscanner.medschl.cam.ac.uk/?query=GATM+&catalogue=mQTL&p=1e-5&proxies=None&r2=0.8&build=37
22_24893867_G_A	UPB1 (hsa:51733)	3-Ureidopropionate (C02642)	1.149e-09	0	No rsID found, PMID:28263315, http://www.phenoscanner.medschl.cam.ac.uk/?query=UPB1&catalogue=mQTL&p=1e-5&proxies=None&r2=0.8&build=37
4_69444550_C_A	UGT2B17(hsa:7367)	sn-Glycerol 3-phosphate(C00093)	2.93e-05	6	No rsID found, PMID: 27005778 (Total phosphoglycerides)http://www.phenoscanner.medschl.cam.ac.uk/?query=UGT2B17&catalogue=mQTL&p=1e-5&proxies=None&r2=0.8&build=37
13_42683873_C_T	DGKH(hsa:160851)	Tetradecanoyl-CoA∗(C02593)	2.72e-05	8	rs75732304 PMID: 21886157 (2-tetradecenoyl carnitine/gamma-glutamylmethionine∗)http://www.phenoscanner.medschl.cam.ac.uk/?query=DGKH&catalogue=mQTL&p=1e-5&proxies=None&r2=0.8&build=37

Column 1: SNP information formatted as CHROMOSOME_POSITION_ALLELE1_ALLELE2 in hg19. Column 2: Gene symbol found the in the KEGG ID entry. Column 3: Metabolite common name found the in the KEGG ID entry. Column 4: p value of the association between the SNP and the metabolite. Column 5: SRD annotation of the gene-metabolite pair using KEGG graph hsa01100. Column 6: Miscellaneous information for the association: rsID if available; PMID of the publications reporting association between the gene, or the SNP with the associated metabolite; link to the query used in Phenoscanner website. Abbreviations: ID = Identifier; SNP = Single-nucleotide polymorphism; SRD = Shortest Reactional Distance, PMID = PubMed Identifier. ∗ CoA are representatives of the measured carnitines.

## Discussion

The identification of an association between an SNP and a metabolite is usually supported solely by the p values of the statistical test without consideration for the known metabolic pathways. Although the possibility of false positive hits is well understood among geneticists following up on mGWAS results, the fact that reporting of gene-metabolite pairs using only statistical significance can lead to an incomplete list of associations is less discussed. In this study, we demonstrate how a simple metric called shortest reactional distance (SRD) can be useful for the reporting and the *ad-hoc* annotation of gene-metabolite pairs for several p values acceptance cut-offs. By using previously published mGWAS, we have shown that the SRD is a metric capable of retrospectively identifying a number of false negatives. The datasets we used in our study involved different genomics and metabolomics protocols, different preprocessing strategies, different sets of genetic variants (genotyped, imputed, and exclusive to exons) and of metabolites, different ethnicities (from European, African, and Middle Eastern ancestries), disease-based and population cohorts, illustrating the wide range of contexts in which our annotation is applicable.

PathQuant, the package developed to compute the SRD metric in this study, is not the only package available that computes the shortest path metric between genes and metabolites. Similar to PathQuant, MetaboSignal^13^ is an R package based on the same mathematical criterion of shortest path metric and also uses metabolic pathways from the KEGG database. There are, however, important differences between these two methods. MetaboSignal combines both metabolic and signaling pathway maps. While this can provide novel information about the interaction between genes and metabolites that goes beyond the known enzymatic reactions and pathways, it modifies the original topology of the curated pathways (signaling and metabolic). These changes are not necessarily supported by biological data, which is crucial to ensure their validity, as emphasized by Dumas et al.^17^ Thus, for our assessment of the potential of SRD annotation in mGWAS, we favored a simpler approach that focuses only on curated metabolic pathways, by converting a metabolic pathway map from KEGG into a graph of biochemical reactions with metabolites as nodes and genes as edges. PathQuant computes SRD values from any given list of gene-metabolite pairs using any given metabolic pathway graph in KGML format thus keeping the original and curated topology of the metabolic pathways reported by KEGG. Of note, a direct comparison of the shortest path metrics calculated by PathQuant and MetaboSignal on the list of gene-metabolite pairs of the mGWAS investigated here could not be performed, as the KEGG overview pathway graph we used with PathQuant is not accepted as input by MetaboSignal. Indeed, MetaboSignal computes its shortest path metric using a custom graph that is built from a pre-selected list of signaling and metabolic pathways.

The computation of the SRD metric with PathQuant within KEGG graphs leads to several possible values: numerical, infinite, or NA. Having a numerical annotation to a pair which is based on known pathways leads to a better understanding of the underlying biology of an association. Indeed, we have shown that SRD values decrease as statistical significance of gene-metabolite pairs increases: this negative correlation between the level of significance and SRD values suggests an enrichment of biologically relevant pairs as the p value decreases, thus making SRD a promising annotation metric to improve mGWAS reporting. We defined two categories for numerical values (short SRD: 0 < SRD ≤ 8; large SRD: SRD > 8), which have been derived from the topological properties of the KEGG overview graph (ID: hsa01100) as this graph contains all curated human biological reactions. The annotation of a pair with a small SRD suggests that the gene, its expression levels or the protein it codes for, may have a direct influence on the associated metabolite concentration. This is illustrated by the statistical difference between the SRD values of mGWAS pairs and the null distribution of SRD values within the KEGG overview graph and by manual investigation of specific pairs of interest, such as the *CPS1* and L-citrulline finding (SRD = 1) in TK study, the *UMPS* and orotate example (SRD = 0) in HMC study. Moreover, for the SCD study, multiple gene-metabolite pairs annotated with an SRD lower or equal to 8 (Table 2) had already been reported in the literature, such as the *DGKH* and tetradecanoyl-CoA (SRD = 8) or *UGT2B17* and sn-glycerol 3-phosphate (SRD = 6). Despite the number of investigated associations being very small for the R and S+ categories, we observed a decreasing proportion of pairs with values lower than 8 and an increasing frequency of pairs with values greater than 8, as p values increases, in line with the negative correlation observed. These results demonstrate, across all different mGWAS datasets we used, the potential of using the SRD metric with a cut-off of SRD ≤8 in order to identify false negatives hits and prioritize follow up studies and experiments.

Another interesting case is when the SRD value is large, but the p value is highly significant. In this case, a direct influence of the gene on the associated metabolite is less clear. For example, we noticed a large SRD value for the association between the alkaline phosphate (*ALPL*) and the phosphocreatine (SRD = 22; p value = 9.356 × 10^−10^). One possibility is that this association could have been a false negative. However, digging further into the literature, we found that phosphocreatine can, in fact, be the substrate of the enzyme encoded by the *ALPL* gene.^44^^,^^45^ Thus, the true SRD value for *ALPL*-phosphocreatine pair should be 0, and the high SRD value computed is a consequence of the current state of the publicly shared knowledge available on KEGG. This result highlights a limitation of our approach, which is the incompleteness of some metabolic pathways in KEGG. Although the SRD metric leverages existing and known KEGG knowledge, our SRD annotation pipeline of mGWAS results can help identify those cases and could be useful to detect gaps and errors in metabolic network databases. Other cases of SRD >8 for highly significant association have been noted in our work, notably in TK study (Figures 2A, 2C, and 3A). When the path computed in KEGG is accurate and the association is independently replicated, higher values of SRD can identify metabolic pathways involving more indirect gene-metabolite relationships. By themselves, these cases are of interest as they illustrate a violation of the hypothesis that biological proximity between a gene and a metabolite is needed to regulate its level.

An SRD annotation with an infinite or NA value means that there was no path between the gene and the metabolite within the chosen graph, because they are not connected (infinite value) or because one of the two entities (or both) are missing from the queried graph (NA value). In most cases, there likely exist no biological paths between these entities, but if the association is highly significant, it could reflect actual gaps within the graph again. Investigation of enzymes or metabolites with an unusual number of infinite or NA values may lead to more complete metabolic pathway databases. Beyond these potential gaps in KEGG, other limitations of this resource are the metabolic reactions provided do not specify cofactors, enzymatic complexes required for the reaction to happen, and directionality, resulting in a decreasing level of complexity and accuracy of the metabolic pathways. Of note, the KEGG overview graph primarily centers on enzymes, with 1,321 genes encoding for enzymes out of the 1,351 genes implicated in pathway reactions (>97%). Enzymes stand out due to their marked substrate specificity, which distinguishes them from transporters and others type of encoding genes. Here, we focused our analysis on associations that mirror the distinctive specificity within the cascade of enzymes and their associated metabolites, which is why we narrowed down our investigation to SNPs mapped to genes encoding for an enzyme. Thus, computing SRD on other resources for pathway mapping, such as Recon3D, could improve our distance-based annotation pipeline. Indeed, this resource is considered the most complete reconstruction of human metabolism so far.^46^^,^^47^ Furthermore, in contrast to KEGG, Recon3D has the advantage of including more genes encoding transporters, which would allow to increase the breath of SRD computation beyond enzymatic reactions. It also includes information about cellular compartments and the directionality of reactions is known, in contrast to KEGG graphs. Despite these advantages, the available file format (sbml) used in Recon3D to represent metabolic pathways is incompatible with the graph structure we used for KEGG. Given that the goal of this study was to establish if the SRD annotation is useful for mGWAS annotation, a simple representation of metabolic pathways is highly appropriate and it is outside the scope of the present study to implement an SRD metric based on Recon3D. Future implementations on alternative databases should consider two additional desirable characteristics of KEGG when comparing results: first, KEGG is the only database for which pathway maps were built on known reactions from humans and others species, a clear advantage for non-human mGWAS studies^48^^,^^49^; second, KEGG pathway maps are built with an explicit labeling of side compounds definition (such as ATP or H_2_O) from KEGG RPAIRS,^50^ making their removal from curated reactions possible, which eliminates shortcuts created by their over-representation within the graph when computing SRD.^51^

A crucial step for the annotation of gene-metabolite pairs with SRD values is to obtain the KEGG IDs for the genes and metabolites. Although gene names and IDs are highly standardized^52^ there is a lack of uniformed nomenclature for metabolites. In most cases, there are multiple ways to refer to a metabolite, with different studies using different reporting conventions, which complicated the re-analysis of published datasets. For HMC and SCD studies, HMDB IDs^3^ were provided by the authors, which could be easily converted to KEGG IDs using the MetaboAnalyst Convert tool. In the case of the TK study, we performed a manual annotation of the common names to KEGG IDs, but this process was time consuming and required having the relevant expertise. This manual work allowed us to include listed metabolites that did not have KEGG IDs, such as acylcarnitines (see Material and STAR Methods). These metabolites are measured in plasma but arise from intracellular metabolism of their acyl-CoA counterparts, which in contrast to acylcarnitines do not cross the plasma membrane. Based on the known direct link between the acylcarnitines and the acyl-CoAs we have used the CoA counterparts to represent these metabolites, which do have KEGG IDs. The increasing quantity of released studies involving metabolomics in the literature encourages the community to release new standards to refer metabolites, such as simplified molecular-input line entry system,^53^ coordination of standards in metabolomics,^54^ and IUPAC international chemical identifier.^55^ These efforts for standardization of metabolites naming, and the reporting improvements they allow, are leading to a promising leap for the field, as this will result into better links being made between novel findings and already published data.

In practice, SRD annotations can be a great addition to bioinformatics pipelines in order to reduce the number of potential candidate pairs to follow up on, by prioritizing hits based on curated biological information, as the number of released mGWAS grows. Moreover, PathQuant provides an efficient and quantitative solution to extract information from metabolic pathways and enables non-experts to efficiently leverage their combined genomics and metabolomics data based on a straightforward metric. This fast and automated solution has the potential to become a new standard metric within the mGWAS toolkit, and could help to systematically assess the proportion of false negatives in these studies. Furthermore, this metric could be added within already available user-friendly resources such as mGWAS Explorer^14^ or the recently developed Paired Omics Data Platform.^56^ SRD values could also play a role in designing targeted studies, in a context where extensive mGWAS cannot be done or is not relevant, for example if the research question is about finding the genes or proteins that regulate the levels of a specific metabolite, or conversely, finding which metabolites are regulated by a specific enzyme. In this latter case, it would make sense to focus more specifically on the metabolites showing low SRD values with the targeted enzyme. In the future, we believe that extending the implementation by using other appropriate resources for pathway mapping would likely improve coverage and enhance the value of the SRD metric in mGWAS annotation pipelines.

### Limitations of the study

Our usage of the SRD metric here was meant to demonstrate its utility for annotating mGWAS results, but there are limitations and alternative considerations that need to be taken into account to improve this workflow. We fixed the cut-off to 8 for defining a small SRD value, but depending on the study goals and context, different SRD thresholds could be used. By considering graph complexity, future users can derive a relevant threshold suitable to their study specificities, as well as a pre-selection of pathways on which to compute their SRD values, based on metabolites and disease investigated. We also recognize that identifying potential causal genes linked to SNPs, and thus the appropriate set of gene-metabolite pairs, is a challenge extending beyond the scope of mGWAS alone. In the context of our study, we have used SNP-based summary statistics to retrieve loci, but more sophisticated mapping approaches in mGWAS, such as gene-based association tests, hold the promise of extracting a more precise set of gene-metabolite pairs, which will, in turn, contribute to a more comprehensive understanding of the applicability of SRD in annotating mQTLs. Using the prevailing approach of associating SNPs to the nearest gene and testing different genomic distance intervals, our analyses suggest that the 10 kb interval is a suitable interval to uncover close relationships in metabolic pathways, but extending the queried region to 50 kb led to the inclusion of relevant pairs with small SRD values and could be appropriate to consider in future studies. We further extended our gene-metabolite mapping strategy to a mapping approach involving eQTLs to uncover supplementary pairs, which had, intriguingly larger SRD in average than pairs found by mapping genes within 50 kb of the associated SNP. This might suggest the limitations of eQTL detection power in capturing closely associated mQTLs within metabolic pathways. Alternatively, it may reflect the incomplete incorporation of novel eQTL-based relationships into current metabolic pathways, making SRD and eQTL annotations complementary.

### Conclusions

We consider this work as a proof of concept of the benefits of using shortest reactional distance metrics for annotating mGWAS results based on a model representation of the human metabolism provided by the KEGG database. These metrics can also be used as a tool for metabolic databases in order to more easily identify gaps within current metabolic pathway graphs by using new information provided by mGWAS. In this multi-omics era, we anticipate that large scale studies looking for associations between genomics, proteomics and metabolomics signals will soon become a new standard, as illustrated by recent studies in mice and humans,^57^^,^^58^^,^^59^ resulting in additional protein-metabolite pairs, for which the computation of SRD values may add great value.

## STAR★Methods

### Key resources table

**Table:** 

REAGENT or RESOURCE	SOURCE	IDENTIFIER
**Deposited data**		

TK	https://doi.org/10.1038/ng.2982	https://static-content.springer.com/esm/art%3A10.1038%2Fng.2982/MediaObjects/41588_2014_BFng2982_MOESM50_ESM.xlsx
TK None	https://doi.org/10.1038/ng.2982	Summary stats without p value cutoff: https://metabolomics.helmholtz-muenchen.de/gwas/
HMC	https://doi.org/10.1038/s41467-017-01972-9	Supplementary 7, https://static-content.springer.com/esm/art%3A10.1038%2Fs41467-017-01972-9/MediaObjects/41467_2017_1972_MOESM9_ESM.xlsx
SCD	https://doi.org/10.3324/haematol.2022.281180 and https://doi.org/10.1016/j.bcmd.2020.102504	https://github.com/HussinLab/PathQuant/blob/main/Publication/SCD_study_download_link.txt
eQTLGen Consortium	https://doi.org/10.1038/s41588-021-00913-z	https://www.eqtlgen.org/
Supplementary data	To be submitted	Available at www.github.com/HussinLab/PathQuant/Publication/References DOI on Zenodo will be provided upon acceptance.

**Software and algorithms**		

PathQuant	This study	www.github.com/HussinLab/PathQuant/https://doi.org/10.5281/zenodo.10023050
bedtools version v2.30.0	https://doi.org/10.1093/bioinformatics/btq033	www.github.com/arq5x/bedtools2/releases/tag/v2.30.0
R statistical software 3.4.4	R Foundation for Statistical Computing	www.r-project.org/

### Resource availability

#### Lead contact

Further information and requests should be directed to and will be fulfilled by the lead contact, Julie Hussin (julie.hussin@umontreal.ca).

#### Materials availability

This study did not generate new materials.

#### Data and code availability

• Data: Source data statement. This paper analyzed existing, publicly available summary mGWAS data for the TK and HMC studies. The SCD study data comes from genomic and metabolomics of two previously published studies. Raw data can be made available upon request to GL. Summary statistics of SCD study will be be shared by the lead contact upon reasonable request. All additional files necessary to reproduce the reported results of this paper are available at: www.github.com/HussinLab/PathQuant/Publication/References.
• Code: PathQuant source code is available at www.github.com/HussinLab/PathQuant/.
• Any additional information required to reanalyze the data reported in this paper is available from the lead contact upon request.

### Method details

#### Overview of the Metabolite genome-wide association studies datasets

In this study we used three different mGWAS datasets. The TK study refers to an mGWAS^11^ previously performed on 7,824 participants (plasma or serum samples) from the TwinsUK cohort^19^ and the KORA study.^20^ The mGWAS was carried out on 2.1 million genotyped SNPs and 529 metabolites (assessed by targeted and untargeted metabolomics). The authors reported 299 SNP-metabolite (or ratio of metabolites) significant associations (at cut-off of p ≤ 1.03 × 10^−10^ for metabolite concentrations and p ≤ 5.08 × 10^−13^ for pairwise metabolite ratios) involving 187 unique metabolites and 145 loci, annotated as 132 causal genes.^11^ The TK study also made available mGWAS output files from METAL software^60^ involving 486 metabolites without any p value cut-off [www.metabolomics.helmholtz-muenchen.de].

The HMC study refers to an mGWAS^21^ previously performed on 614 Qatari participants (plasma samples) from the Hamad Medical Corporation. The mGWAS was carried out on 1.6 million imputed exome variants and 826 metabolites (assessed by targeted and untargeted metabolomics) and reported 3,127 significant associations (at cut-off of p ≤ 2.2 × 10^−10^ for both metabolite concentrations and pairwise metabolite ratios) in 21 locus-metabolite pairs. The suggestive association results were also provided to the community, reporting all associations with cut-off of p ≤ 1.4 × 10^−7^ (Supplementary Data 7 in^21^), which include 6517 SNP-metabolite (or ratio of metabolites) pairs with available p values.

In the SCD study, an mGWAS was performed here using genetics and metabolomics data published in different studies^22^^,^^23^ on a total of 651 SCD patients (plasma samples) including 401 individuals of African ancestry in the Genetic Modifier (GEN-MOD) cohort and 250 African-American individuals from Southwest USA in the Duke University Outcome Modifying Genes (OMG) cohort. Metabolomics profiling was performed at the Broad Institute, and appropriate statistical modelling was used to account for residual batch effects.^61^ Briefly, for association testing, 128 metabolites were profiled using a targeted approach in 651 plasma samples from SCD patients. Metabolites were inverse normal transformed, adjusting for age and sex. We then generated summary statistics for each cohort individually in a linear regression model, accounting for relatedness using a kinship matrix as implemented in rvtest (v. 20171009)^62^ in GEN-MOD. The software SNPTEST^63^ was employed in OMG to generate cohort-specific summary statistics. We then meta-analyzed the effect size estimates and standard errors from GEN-MOD and OMG using METAL.^60^ After the pre-processing step, 6 million SNPs were kept.

For the TK study, we downloaded the file named “NIHMS58114-supplement-2.xlsx” from the supplementary section of the publication. This first file contains only stringently associated pairs. We make a distinction between the TK and the “TK None” datasets as they do not use the same files from the original publication. The TK None is referring to the GWAS summary stats, without any p value cut-off, directly downloaded from here: https://metabolomics.helmholtz-muenchen.de/gwas/index.php?task=download. We downloaded and merge the files named: shin_et_al.metal.out.tar.gz, shin_et_al.xeno.metal.out.tar.gz. As the positions were coming from NCBI Build 36, we performed a lift over to hg19 in order to have same build across all different studies.

#### Mapping shortest reactional distances (SRD) onto KEGG

To compute the SRD metric, the R package PathQuant was developed [available at: www.github.com/HussinLab/PathQuant]. PathQuant converts a metabolic pathway map into a graph of biochemical reactions with metabolites as nodes and genes as edges (Figure 1). Briefly, PathQuant takes as input a list of gene-metabolite pairs as pairs of KEGG identifiers (IDs) and a list of metabolic pathways (e.g., “hsa01100”, referred herein as KEGG overview graph, a global concatenation of multiple distinct pathways). PathQuant then uses a KEGG XML file format (KEGG Markup Language, KGML), downloaded using the KEGG API [www.kegg.jp/kegg/rest/keggapi.html] to build the most up-to-date KEGG undirected graphs. Next, PathQuant computes the SRD path between a gene and a metabolite from a given pair. The SRD values are obtained using the breadth-first search Dijkstra algorithm.^64^ PathQuant outputs a text file containing genes and metabolites classification, Enzyme Commission number (EC), KEGG Brite IDs, KEGG IDs of metabolic pathways for the SRD computation, and the SRD values for all pairs. PathQuant also allows the visualization of SRD values annotation in a heatmap, leading to a better identification of potentially interesting hits.

#### Extracting and annotating gene-metabolite pairs from mGWAS summary statistics

Using *bedtools version v2.30.0*,^65^ we annotated each SNP to genes with KEGG IDs, using a custom bed file: we downloaded gene coordinates for human genome build GRCh37.p13 from NCBI (NCBI Homo sapiens Updated Annotation Release 105.2020/10/22, gff format) and modified it to add 10kb upstream and downstream for each of the 26,105 genes with KEGG IDs, as well as broader intervals of 50kb, 500kb and 1Mb. We also mapped additional gene-metabolite pairs using eQTL annotations from the eQTL Gen consortium with a False Discovery Rate (FDR) threshold ≤5%. Because we focused on pairs involving genes encoding enzymes, which are the most represented genes in the KEGG database, we only annotated SNPs with KEGG IDs classified as enzymes within the KEGG Brite database by using the Brite enzyme code “BR:hsa01000”, leading to a subset of 4,049 enzymes/26,105 genes. For metabolites, if available, we used the provided IDs, either KEGG IDs directly or HMDB IDs. For metabolites with HMDB ID the list was queried to *Metaboanalyst*^66^[www.metaboanalyst.ca/faces/upload/ConvertView.xhtml] to get the corresponding KEGG IDs. For metabolites without KEGG or HMDB IDs provided, KEGG IDs retrieval was achieved automatically by parsing the common names into the most standard KEGG name format, and then queried the metabolite names with new format to MetaboAnalyst. Additionally, specific metabolites were manually treated: acylcarnitines without KEGG IDs were swapped to their acyl-CoA counterparts when available in KEGG. For each gene-metabolite pair within a dataset (TK, HMC and SCD study), only one mGWAS p value was kept, which is the minimum p value obtained for the association between any SNP annotated to the gene and that specific metabolite. Based on that p value, each pair is annotated as genome-wide significant, suggestive, or non-significant: genome-wide p value cut-offs were derived from a standard GWAS Bonferroni correction approach (dividing by the number of tested metabolites). Suggestive significance cut-offs, were obtained graphically from quantile-quantile plots (QQplots) of minimum p values for the variants-metabolite pairs of corresponding study. Specifically, we determined the value on the QQplot’s x axis where the slope starts to increase drastically. This threshold represents the point at which the expected p values deviate from the diagonal line, denoting a departure from the null hypothesis. This departure indicates that p values are smaller than what would be expected by chance alone, resulting in a selection of SNPs with an elevated probability of being associated with the measured metabolites.

#### SRD null distribution and investigation of candidate gene-metabolite pairs

To obtain a null distribution of SRD values from the KEGG overview graph (hsa01100), we gathered all different KEGG IDs for both metabolites and genes found within the graph, and then ran PathQuant on all possible pairs (Supplementary File 1). The KEGG overview graph version (KGML v0.7.2 file) includes 1,351 genes and 2,889 metabolites, generating a total of 3,903,039 pairs for which we computed the SRD values, referred herein as the KEGG overview graph’s SRD null distribution. The first quartile of this distribution is used as the threshold to categorize any pair with a close or far biological relationship label. This threshold was determined because 25% of the smallest values are below half of the mean, leaving 75% as a representative sample of distant relationships between genes and metabolites. We also performed a manual investigation of candidate gene-metabolite pairs (Tables 2 and S1 containing the investigation overview) by extracting the corresponding rsID using dbSNP annotation of UCSC Genome Browser,^67^ searching for published associations of the metabolite with (1) the SNP rsID within GWAS Catalogue and/or with (2) the gene symbol within PhenoScanner^6^ (with parameters: cut-off p value = 1e-5, cut-off r^2^ = 0.8, build = 37).

#### Running time of PathQuant package

The overview’s null SRD distribution is the largest distribution of tested gene-metabolite pairs in this study, involving millions of pairs (Figure S7). In order to compute the SRDs for the null distribution, as we recommend, we parallelized the query by generating different queries of 20,000 pairs. Here to show the running time of PathQuant, influenced by the query size, we selected randomly: 1, 10, 100, 1000, 10000, and 20000 gene-metabolite pairs from the null distribution and ran PathQuant using the get.srd() function within the largest graph of the KEGG database (“overview”, hsa01100). In all following experiments, to address the variability of the different running times we reproduced the experiments 10 times, we used PathQuant on the Compute Canada servers, with a sbatch job allocation of 64G memory, and 1 CPU per task.

The Figure S8A shows the variability of the running time of PathQuant (in seconds) for different randomly selected queries of different sizes. We show that the running time of PathQuant is fast enough for users interested in a few pairs but also fast enough for users that will need to compute it for large queries with a running time close to one hour. In order to show the influence of PathQuant running time based on the query size plus the complexity of the involved graph, we performed the get.srd() function with randomly selected queries of size 1, 10 and 100 only. The complexity of a graph can be defined by different metrics. Here, we used the “diameter” (D) metric as it involves the notion of shortest distance. To find the diameter of a graph, we first find the shortest path between each pair of nodes, the largest value of any of these shortest paths is the diameter of the graph. We selected three different graphs, involving a range of different diameters, the graphs hsa01200, hsa00020, hsa01100 with D = 10, 20, 66 respectively. In the Figure S8B, we can observe per graph, the influence of the PathQuant’ running time (in seconds) for different query sizes. Firstly, per graph, we can see the increasing running time for an increasing query size. Finally, we can also observe the increasing running time for the same query size across the different graph complexities while still being fast enough for the most complete KEGG graph and decent query sizes.

### Quantification and statistical analysis

Statistical and data analyses were performed with R. Statistical analyses details can be found in the methods section that describes the experiment, or corresponding Results section.

## Supplemental information

Document S1. Figures S1–S8

Table S1. Manual annotation of S+/S- hits, related to Figure 4, STAR Methods

Table S2. Genome-wide significant associations from the SCD study, related to STAR Methods, Figure S6
