# Supplementary material for: Gene-metabolite annotation with shortest reactional distance enhances metabolite genome-wide association studies results
Source: iScience. 2023 Nov 14;26(12):108473. doi: 10.1016/j.isci.2023.108473 (PMC10709128; doi:10.1016/j.isci.2023.108473)
Supplement: Document S1. Figures S1–S8 [file mmc1.pdf]

iScience, Volume *26*

## Supplemental information

# Gene-metabolite annotation with shortest reactional distance enhances metabolite genome-wide association studies results

**Cantin Baron, Sarah Cherkaoui, Sandra Therrien-Laperriere, Yann Ilboudo, Raphaël Poujol, Pamela Mehanna, Melanie E. Garrett, Marilyn J. Telen, Allison E. Ashley-Koch, Pablo Bartolucci, John D. Rioux, Guillaume Lettre, Christine Des Rosiers, Matthieu Ruiz, and Julie G. Hussin**

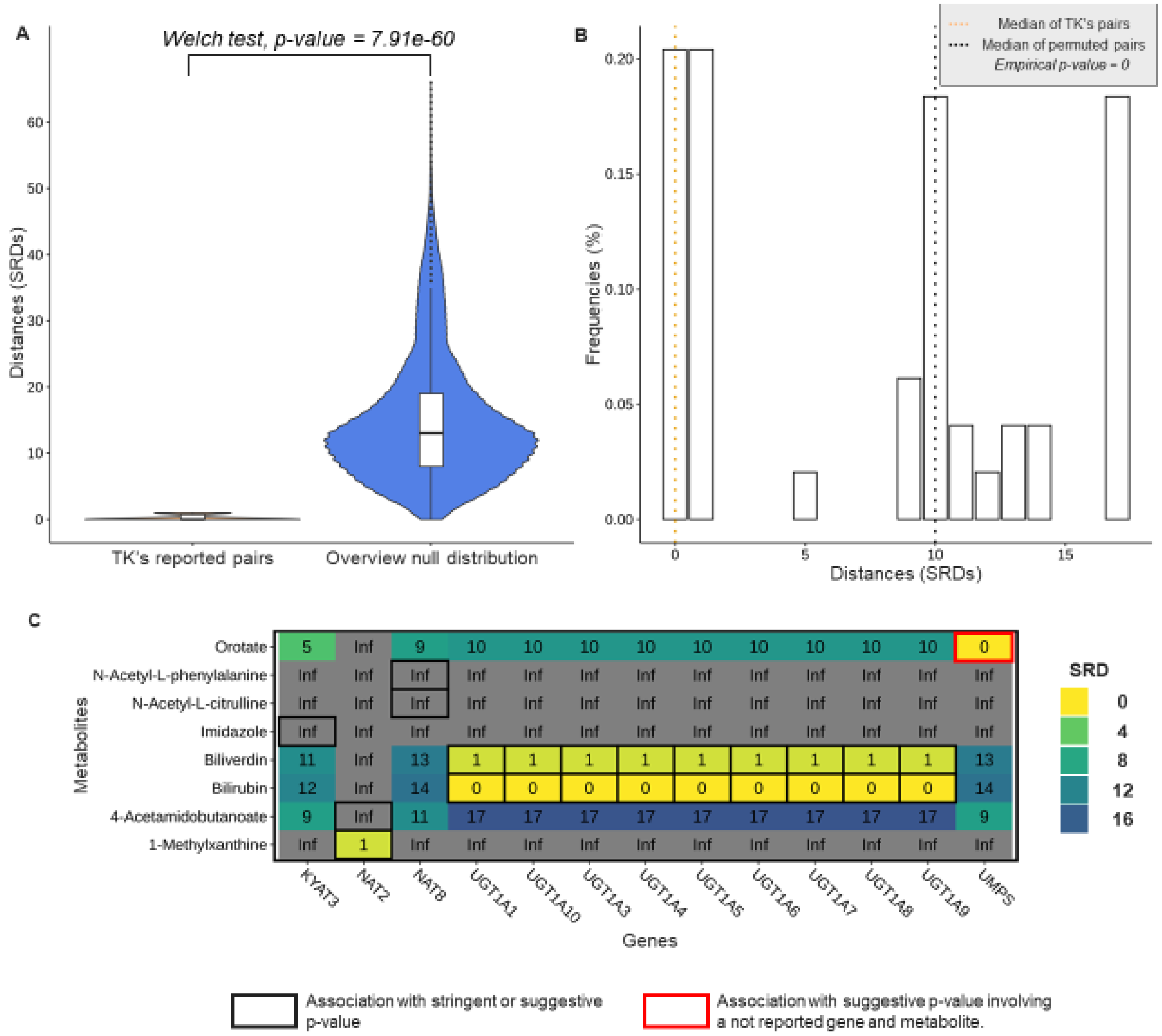


**Supplementary Figure 1. SRD of stringently and suggestive associated gene-metabolite pairs in the HMC study, related to Figure 1.**
(A) Comparison of SRD annotations for reported genome-wide significant associations from the HMC study (orange) and the distribution of all SRD values within KEGG overview graph (hsa01100) (B) Distribution of SRD values computed from permuted gene-metabolite pairs from HMC study, with a median SRD of 10 (black dotted line). The median SRD of 0 (orange dotted line) represents an empirical p-value of p=0. (C) Heatmap representing all genes and metabolites included in reported stringent and suggestive associations from the HMC study. The 24 mapped gene-metabolite associations are enclosed: a black box indicates a reported stringent or a suggestive pair for which either the gene, the metabolite or both have been reported in the dataset (n=23) while a red enclosed box indicates a suggestive pair for which the gene and the metabolite have not been reported in the study (n=1).

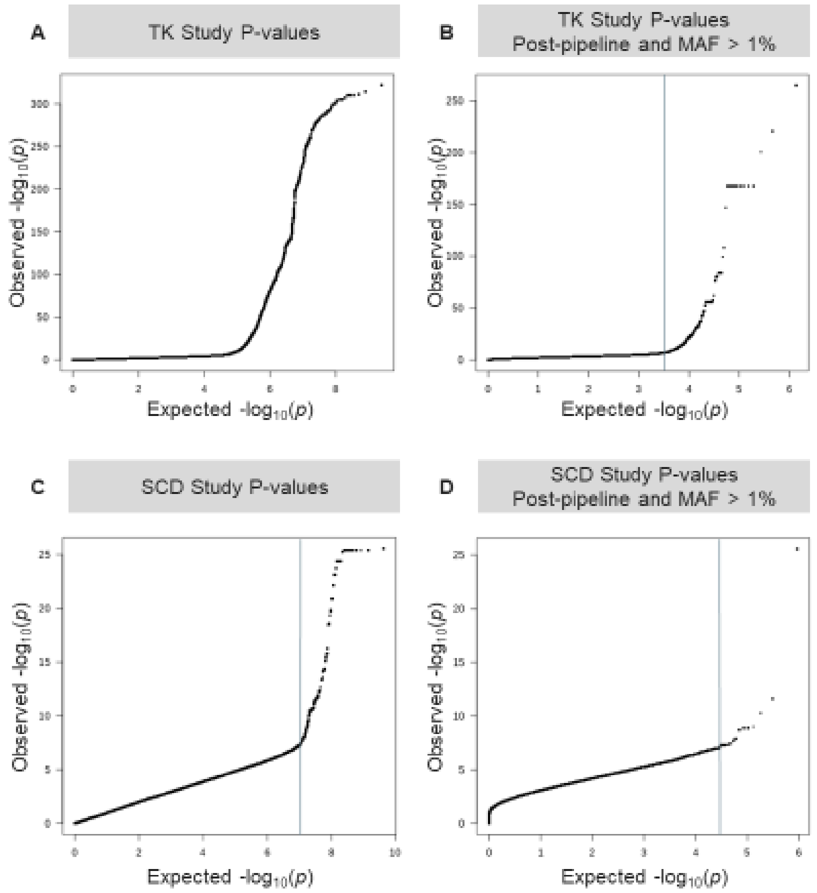


**Supplementary Figure 2. QQplots for the TK and SCD studies, related to Figure 2, Figure 3, Figure 4.**

(A/C) QQplots using all p-values from mGWAS files available, before any pre-processing steps for TK study (A) and for the SCD study (C). (B/D) QQplots using p-values at the end of the pipeline (See STAR Methods) for the TK study (B) and for the SCD study (D). Vertical lines indicate the graphically determined cut-offs we used in our Results section. Abbreviations: MAF = Minor Allele Frequency, QQplot = Quantile-Quantile plot.

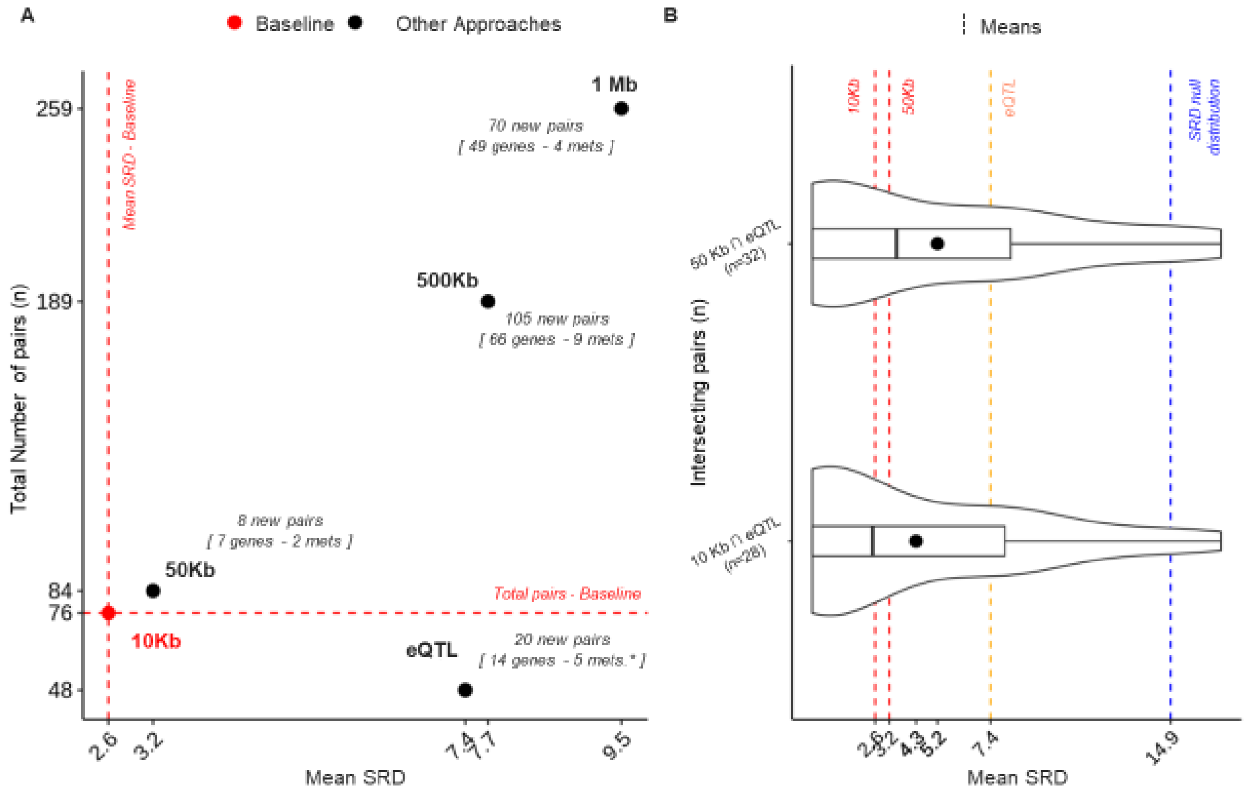


**Supplementary Figure 3. Expending the number of pairs for the TK Study by using alternative SNP mapping approaches, related to STAR Methods.**

(A) Scatterplot of total pairs (y-axis) against mean SRD (x-axis), bold text indicates SNP mapping approaches. Red dashed vertical and horizontal lines mark baseline values from the 10kb approach. News pairs are labeled with gene and metabolite counts in brackets (B) Violin-box plots of SRD values for intersection pairs. The mean SRD is indicated by the black points. Dashed vertical lines mark the mean SRD for 10kb, 50kb, eQTL, and the null distribution, respectively. Abbreviations: eQTL = expression Quantitative Trait Loci, Mets=metabolites.

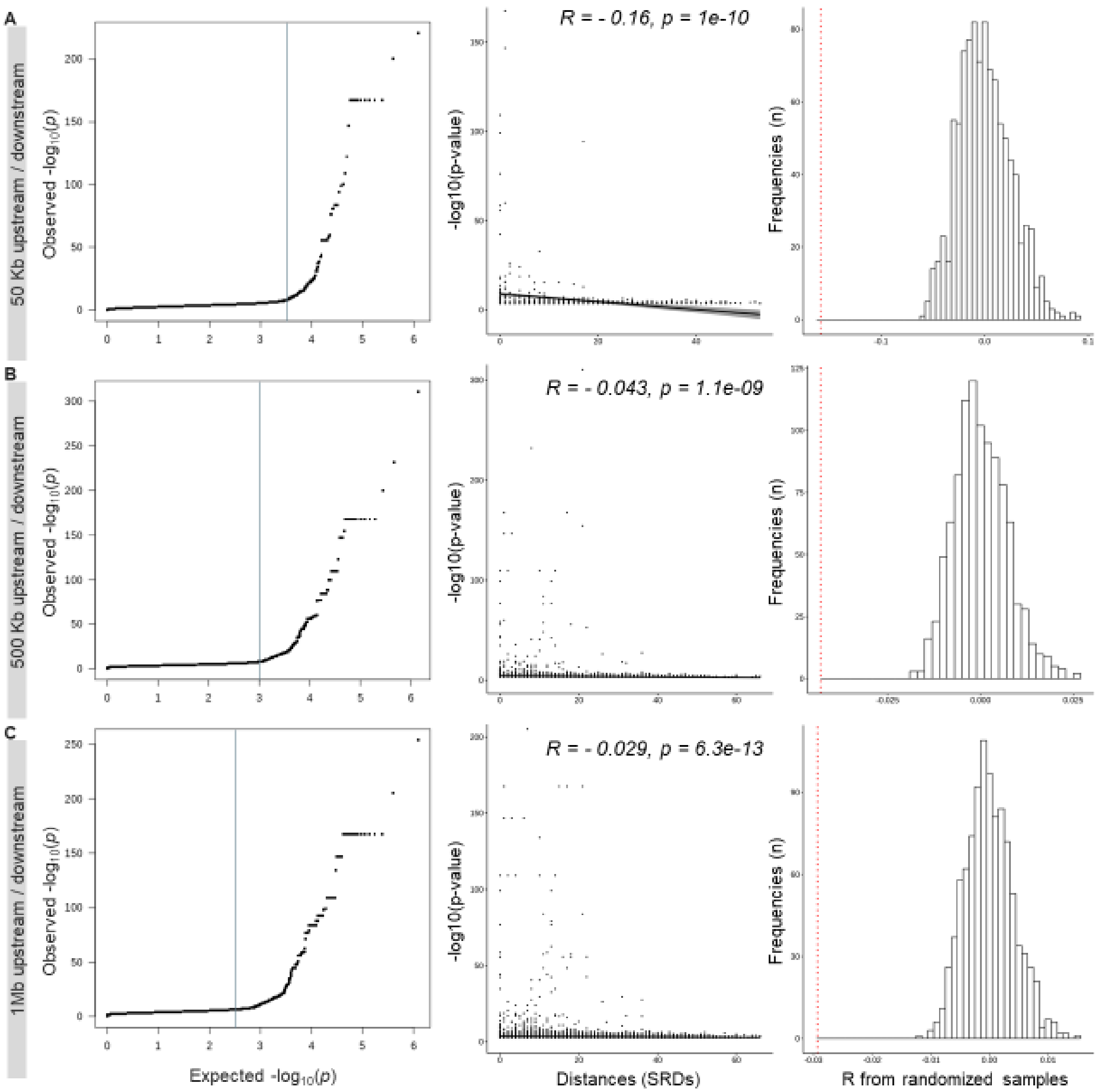


**Supplementary Figure 4. Q-Q and correlation plots for the TK study with the variation in the SNP mapping range, related to Figure 3, STAR Methods.**

(A/B/C) Each panel corresponds to different range for SNP mapping, 50 kb (A), 500 kb (B), 1 Mb upstream and downstream. Left-plots: QQplots using p-values at the end of the pipeline (See STAR Methods). Center-plots: Correlation plot between the –log10(p-values) and SRD values for gene-metabolite pairs. Right-plots: Correlation computed on permuted data (N=1000) to take into account the graph structure allowed computation of empirical p-values, with the true correlation coefficient presented (dotted red line). Solid vertical lines indicate the graphically determined cut-offs we used, dotted red lines indicate the true correlation coefficient (noted as "R" on the left). Abbreviations: QQplot = Quantile-Quantile plot.

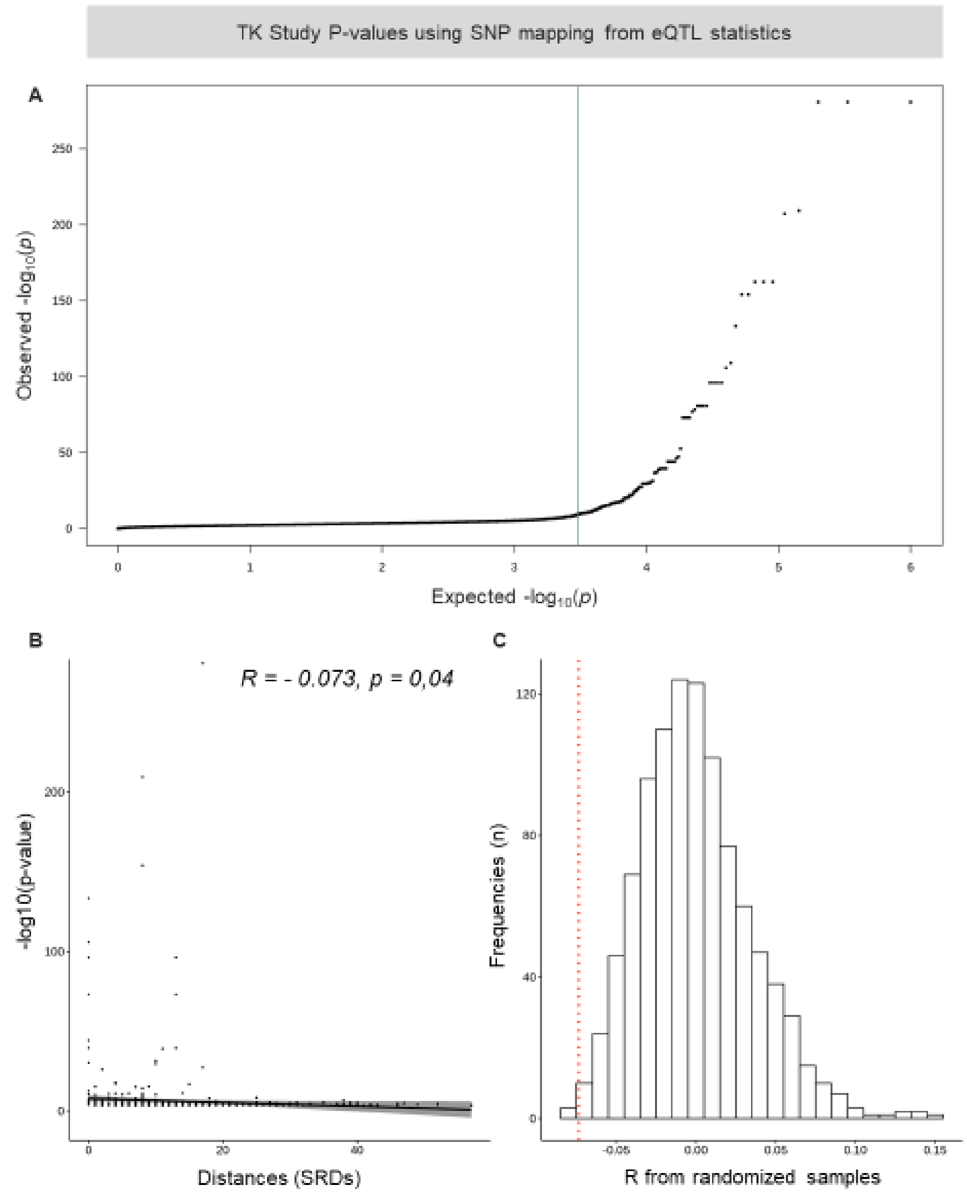


**Supplementary Figure 5. Q-Q and correlation plots for the TK study with the SNP mapping from eQTL statistics, related to Figure 3, STAR Methods.**
(A) QQplot using p-values at the end of the pipeline with the SNP mapping from eQTL statistics (See STAR Methods, Supplementary Material). (B) Correlation plot between the –log10(p-values) and SRD values for gene-metabolite pairs. (C) Correlation computed on permuted data (N=1000) to take into account the graph structure allowed computation of empirical p-values. Solid vertical line indicates the graphically determined cut-offs used, dotted red lines indicate the true correlation coefficient (noted as "R" in B). Abbreviations: QQplot = Quantile-Quantile plot.

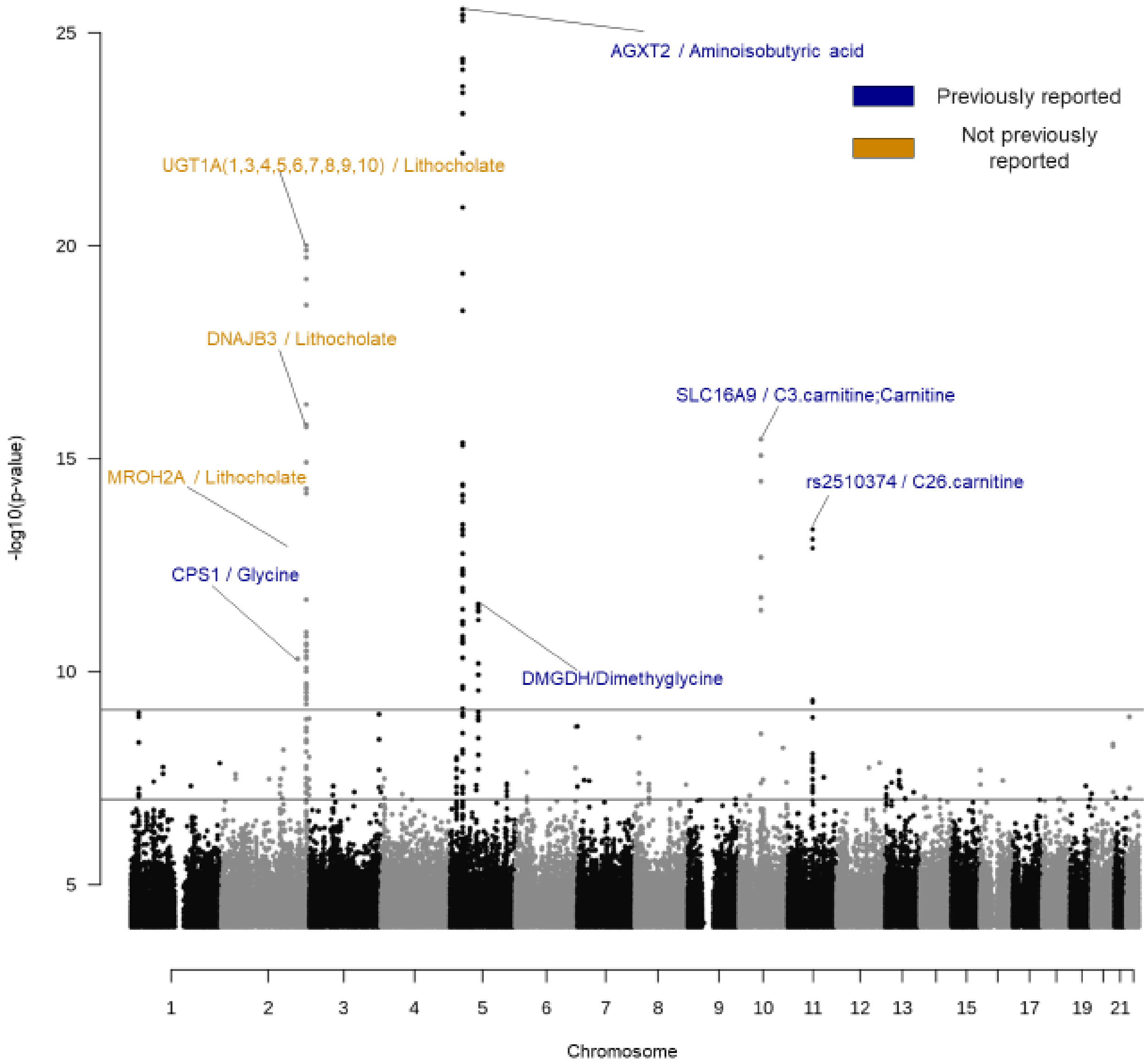


**Supplementary Figure 6. Manhattan plot of the SCD study, related to Figure 4.**
Manhattan plot of the SCD study with a p-values below $1x10^{-4}$ cutoff, each dot represents an association between a variant and a metabolite. Black and white is an alternating color palette for chromosomes. The first line from plot bottom (dark plain) indicates the genome-wide threshold at $1x10^{-7}$, and the second line (light plain) at 7.8125x10-10, indicates the first threshold corrected by Bonferroni (See Methods). Top hits for each mapped gene above the Bonferroni threshold are labelled with two distinct colors: blue if the association has not been previously reported, or orange if the association has been already reported in the literature. Gene symbols are separated to metabolite name(s) by "/" symbol.

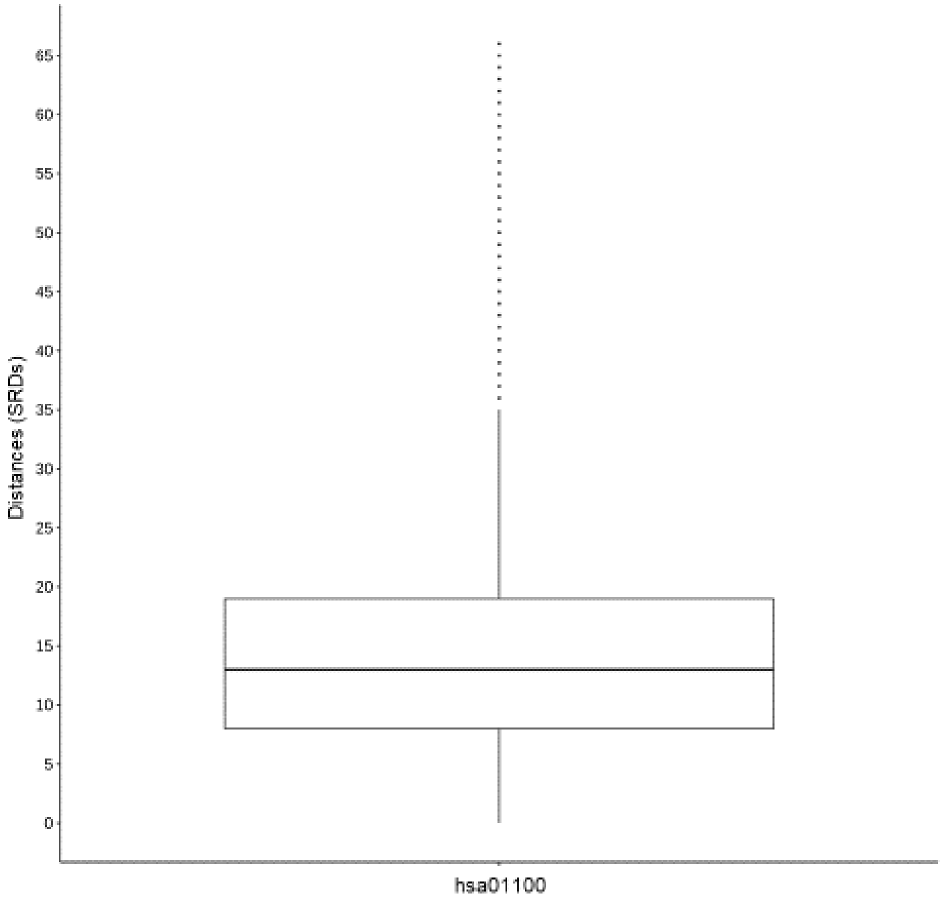


| Min. | 1st Qu. | Median | Mean | 3rd Qu. | Max. |
|---|---|---|---|---|---|
| 0.0 | 8.0 | 13.0 | 14.9 | 19.0 | 66.0 |

**Supplementary Figure 7. Summary statistics of the null distribution of SRD values within the KEGG overview graph, related to STAR Methods.**
Boxplot of the SRD values and table of summary statistics for the null distribution of SRD values within the KEGG overview graph (hsa01100). The mean SRD within this graph is 14.89, with a maximum value of 66. The first quartile (SRD <8) is used as the threshold to categorize any pair with a close or far biological relationship label.

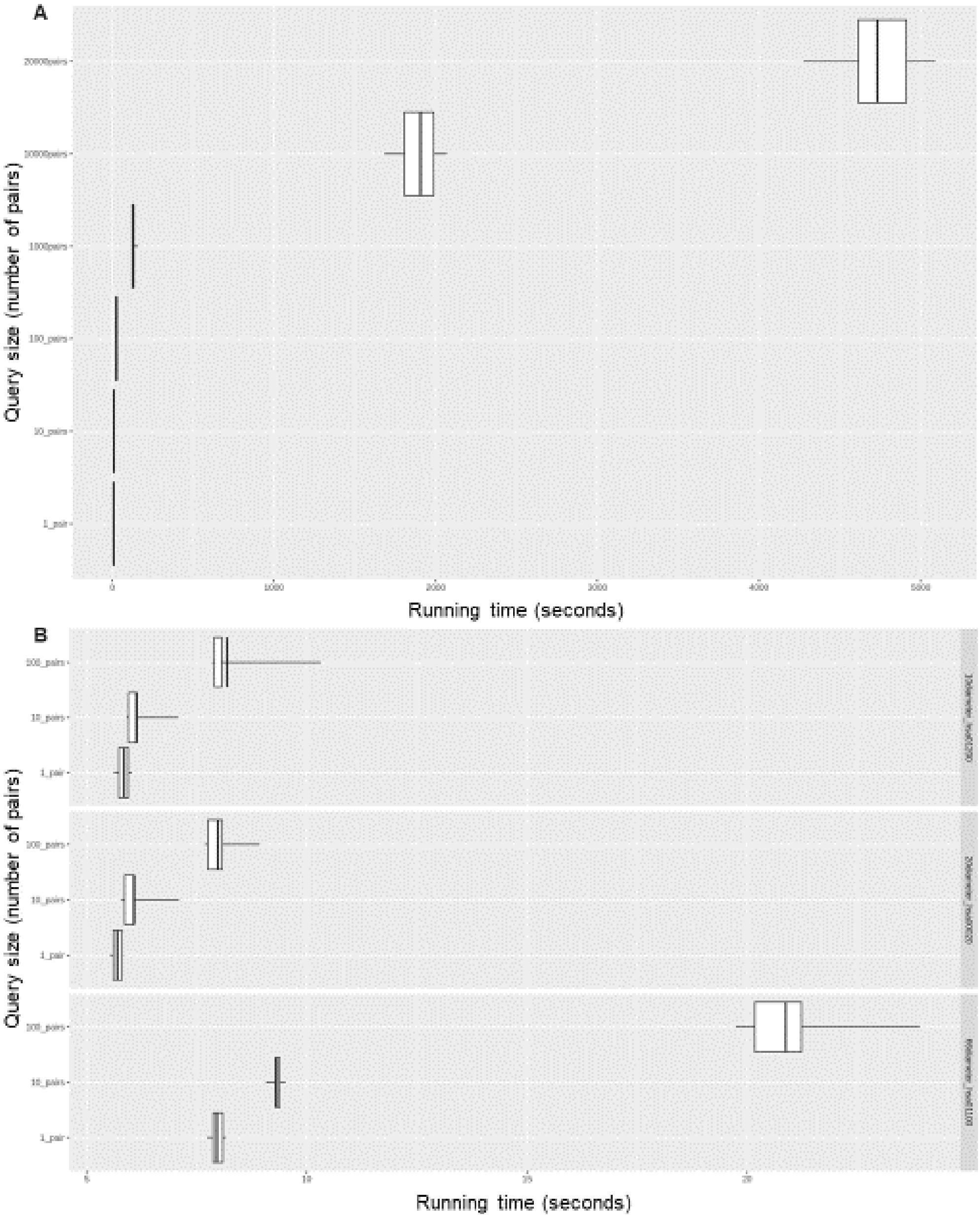


**Supplementary Figure 8. Running time of package PathQuant for different number of tested gene-metabolite pairs, related to STAR Methods.**

(A) Boxplot of running times to obtain the SRD annotation for different number of tested gene-metabolite pairs within the KEGG overview graph (hsa01100), 10 times replication. (B) Boxplot of running times to obtain the SRD annotation for different number of tested gene-metabolite pairs within three graphs of different diameters (from top to bottom: hsa01200, hsa00020, hsa01100), 10 times replication using with a sbatch job allocation of 64G memory, and 1 CPU per task.
